# The evolving role of PSA in prostate cancer screening: revisiting the evidence in the era of personalized medicine

**DOI:** 10.1515/almed-2025-0187

**Published:** 2026-01-28

**Authors:** Xavier Filella

**Affiliations:** Biochemistry and Molecular Genetics Department, Hospital Clinic, Barcelona, Catalonia, Spain

**Keywords:** prostate-specific antigen, prostate cancer screening, risk-adapted screening, PSA assay variability

## Abstract

Prostate-specific antigen (PSA) is a widely available and analytically robust biomarker, but its role in prostate cancer (PCa) screening remains controversial due to concerns regarding limited specificity, overdiagnosis of indolent tumors, and the downstream risk of overtreatment. This review provides a critical analysis of the main randomized trials, systematic reviews, and meta-analyses that have shaped current recommendations against PSA-based screening, highlighting the substantial methodological heterogeneity that complicates interpretation of aggregated outcomes. Key limitations identified across studies include variability in PSA thresholds and screening intensity, insufficient follow-up in some cases to detect long-term mortality benefits, non-comparability of PSA assays, and extensive contamination of control groups – particularly in the PLCO trial – which undermines estimates of screening efficacy. Recent updates from the ERSPC and CAP trials show that mortality reductions associated with screening increase with longer follow-up, supporting the need to reassess earlier conclusions. Parallel to the evolving evidence base, European initiatives such as the PRAISE-U consortium and several regional pilot programs following the European Council’s recommendations are implementing risk-adapted pathways that combine baseline PSA stratification with multiparametric MRI triage and selective biopsy. These programs aim to minimize overdiagnosis while improving detection of clinically significant PCa and generating real-world evidence for organized screening. Additionally, the variability among PSA assays underscores the need for greater harmonization and standardized reporting. Overall, emerging data suggest that intelligently targeted, risk-stratified PSA screening may offer a more balanced approach than the traditional dichotomy of screening vs. no screening.

## Introduction

Tumor markers comprise a broad group of substances produced directly by neoplastic cells or induced by their presence. By reflecting tumor growth and activity, they provide valuable information regarding the presence, progression, and therapeutic response of malignant neoplasms [1], 2]. Numerous studies have demonstrated their usefulness in monitoring oncology patients, particularly for the early detection of tumor recurrence or assessment of treatment response. However, their role in the diagnosis of cancer has been a recurrent subject of debate. Several authors have cautioned against the excessive and inappropriate use of tumor markers, as this may lead to unnecessary investigations and, consequently, increase both the risk and complexity of clinical management [3], [4], [5], [6].

Within this context, the use of prostate-specific antigen (PSA) as a tool for prostate cancer (PCa) detection has become one of the most controversial examples and continues to generate intense debate in both scientific and clinical settings [7], 8].

In Catalonia, in the northeast of Spain, a 2013 document issued by the Department of Health of the autonomic Government of Generalitat stated that the use of serum PSA testing as a population-based screening method for the early detection of PCa is not recommended, as it provides no benefit in terms of mortality reduction [9]. Similarly, in Spain, the guideline developed by GuiaSalud (2013) -an agency of the National Health System-advised against routine PSA testing in asymptomatic individuals without a first-degree family history of PCa [10]. In the same vein, the Population Screening Committee of the Spanish Ministry of Health, in its 2019 report, concluded that the balance between benefits and risks of PSA-based screening is unfavorable [11].

The Sociedad Española de Médicos de Atención Primaria (Semergen) likewise emphasized the lack of clear scientific evidence supporting population-based PSA screening in asymptomatic patients, mainly due to the risk of associated adverse effects such as overdiagnosis and overtreatment [12]. Consistently, the Sociedad Española de Medicina de Familia y Comunitaria stated that PSA testing should not be recommended as a screening tool for PCa in asymptomatic individuals at average risk [13]. More recently, the Asociación Española de Biopatología Médica–Medicina de Laboratorio, in its 2021 document Decisiones inteligentes desde el laboratorio: de elegir sabiamente a no hacer, asserted that there is no conclusive evidence that PSA-based screening reduces mortality, recommending that the decision to undergo screening should be made by the patient within a shared decision-making process [14].

A similar position was expressed by the American College of Preventive Medicine in 2016, which included among its five choosing wisely recommendations the avoidance of PSA-based PCa screening [15]. This stance was also shared by several contemporary clinical guidelines that did not recommend the routine use of PSA for PCa screening in asymptomatic men, including those issued by the Canadian Task Force on Preventive Health Care (2014), the Royal Australian College of General Practitioners (2016), and the UK National Screening Committee (2018) [16].

## Evaluation of four studies assessing the usefulness of PSA in prostate cancer screening

Since the publication in 2009 of the initial results from the European trial (European Randomized Study of Screening for Prostate Cancer*,* ERSPC) and the North American trial (Prostate, Lung, Colorectal, and Ovarian Cancer Screening Trial*,* PLCO) on PSA-based PCa screening [17], 18], several key studies have been conducted that have shaped the current understanding of the benefits and risks associated with this practice.

**Figure 1:**
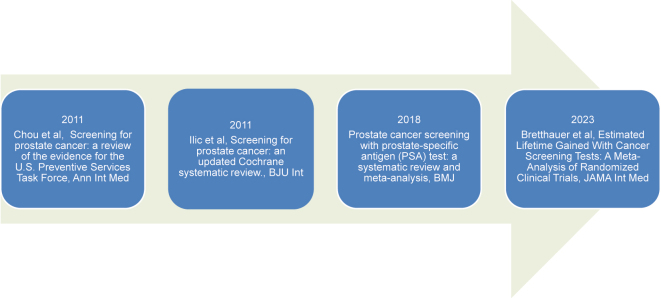
Timeline of studies assessing the utility of PSA in prostate cancer screenin.

In 2011, at the request of the U.S. Preventive Services Task Force (USPSTF), a systematic review of the available evidence on the utility of PCa screening was published [19]. Around the same time, Dragan Ilic and colleagues conducted a Cochrane systematic review on the same topic [20]. Subsequently, Ilic et al. [21] updated their analysis in a 2018 publication, and more recently, Bretthauer et al. [22] estimated life-years gained across various cancer screening programs, including PSA screening for PCa. All of these studies (Figure 1) reached similar, unfavorable conclusions regarding the effectiveness of PSA-based screening and have had a decisive influence on the recommendations issued by multiple scientific societies and health systems against its widespread use.

Chou et al. [19] included six studies conducted in Europe and North America, concluding that PSA-based screening results in a small or no reduction in PCa-specific mortality and is associated with adverse effects resulting from subsequent diagnostic tests and treatments, many of which -they warned- may be unnecessary. This review formed the basis for the 2012 USPSTF recommendation, which took a clear and unequivocal stance against universal PSA-based screening for PCa at all ages [23].

Table 1 [17], 18], [24], [25], [26], [27], [28] summarizes the data included in that review, highlighting significant differences among the trials with respect to the age of the study populations, duration of follow-up, and the PSA threshold considered suspicious, which ranged from 2.5 to 7 µg/L. Among the six included studies, the ERSPC and PLCO trials stand out for their large sample size: 162,387 and 76,693 participants, respectively. However, whereas the European trial reported a median and maximum follow-up of 9 and 14.5 years, respectively, the North American trial provided results with only 7 and 10 years of follow-up.

**Table 1:** Results included by Chou et al. [17] in their systematic review.

Study	Population characteristics	PSA cut-off	Time of follow-up (median/maximum, years)	Differences in prostate cancer–specific mortality
Andriole et al. 2009, PLCO, USA [18]	Men 55–74 aged; intervention group/control group: 38,343/38,350	4 µg/L	11.5/14.8	No at 7 or 10 years
Hugosson et al. 2010, substudy of ERSPC, Goteborg, Sweden [24]	Men 50–64 aged; intervention group/control group: 19,904/9,952	2, 5–3 µg/L	14/14	Yes
Kjellman et al. 2009, area of stockholm south hospital, Sweden [25]	Men 55–70 aged; intervention group/control group: 2,400/24,804	10 µg/L If PSA>7 µg/L: repeat ultrasound	12.9/15.7	No
Labrie et al. 2004, Quebec [26]	Men 45–80 aged; intervention group/control group: 31,133/15.353	3 µg/L	7.9/11	No
Sandblom et al. 2004, 2011, Nörrkoping, Sweden [27], 28]	Men 50–69 aged; intervention group/control group: 1,494/7,532	4 µg/L (only digital rectal examination from 1987 to 1990)	6.25/20	No
Schroeder et al., 2009, ERSPC, Europe [17]	Men 50–74 aged; intervention group/control group: 82,816/99,184	3 µg/L (variations between centers)	9/14.5	Yes

Chou et al. [19] reported that only the studies led by Schröder [17] and Hugosson [24] demonstrated a statistically significant reduction in PCa-specific mortality. It is particularly relevant to contrast these findings with those of Labrie et al. [26], who reported a 62 % reduction in PCa mortality in the screening group (Table 2). That study included 46,484 men aged 45–80 years, of whom 31,133 were invited to undergo PSA screening. However, only 7,348 ultimately underwent testing. In contrast, among the 15,353 men who were not invited, 1,122 also had a PSA measurement performed. The authors compared participants based on whether they actually underwent PSA testing (8,470) or not (39,138), regardless of their original assignment.

**Table 2:** Comparison of the data from the study by Labrie et al. [26] according to the original study and the systematic review published by Chou et al. [17].

Author (analysis	Group	Subjects	Number of deaths
Labrie et al. [26] (according protocol)	Screening with PSA	8,470	10
	No screening (invited and not invited)	39,138	218
Chou et al. [17] (by intention)	Assigned screening	31,133	153
	Assigned control	15,353	75

Chou et al., however, classified participants according to whether they had been invited to screening, consistent with the methodological standard for systematic reviews and meta-analyses in public health policy. This explains why, despite the apparently favorable data reported in the original study, Chou el al did found no difference in PCa-specific mortality. This approach, however, does not account for the fact that 76 % of those invited to PSA screening did not actually undergo the test, and that there was contamination in the control group, with 7.9 % receiving PSA measurements.

The Cochrane review published by Ilic et al. [20] in 2011 reached similar conclusions: PSA-based PCa screening does not significantly reduce disease-specific mortality. That review included five randomized controlled trials [17], 18], [24], [25], [26], [27], among which only the ERSPC trial demonstrated a statistically significant mortality benefit associated with PSA screening.

In 2018, Ilic et al. [21] published an update of their previous review following the release of results from the Cluster Randomised Trial of PSA Testing for Prostate Cancer (CAP), which included 419,357 participants from 573 primary care centers in the United Kingdom [29]. The results of this updated meta-analysis are summarized in Table 3 [26], [29], [30], [31], [32]. As shown in the table, the included studies exhibit a wide heterogeneity with respect to follow-up duration, screening frequency (ranging from a single test to annual testing), cohort size, and the PSA threshold used to trigger further investigation, which varied from 3 to 10 µg/L.

**Table 3:** Results included by Ilic et al. [21] in their meta-analysis.

Study	Population characteristics	PSA cut-off	Screening frequency	Time of follow-up	Differences in prostate cancer–specific mortality
Martin et al, CAP, UK [29]	Men aged 50–69 intervention group/control group: 195,912/219,445	3 µg/L	Single screening	10 years	No
Schroeder et al, ERSPC, Europe [30]	Men aged 55–69 intervention group/control group: 72,891/89,352	3 µg/L	Screening every 2–4 years	13 years	Yes
Labrie et al, Quebec [26]	Men aged 45–80; intervention group/control group: 31,133/15.353	3 µg/L	Single screening	11 years	No
Lundgren et al, Stockholm, Sweden [31]	Men aged 55–70 intervention group/control group: 2,400/25,081	10 µg/L	Single screening	20 years	No
Andriole et al, PLCO, USA [32]	Men aged 55–74 intervention group/control group: 38,340/38,343	4 µg/L	Single screening	15 years	No

Once again, only the ERSPC study [30], now with 13 years of follow-up, showed a statistically significant incidence rate ratio below 1 (0.79). As in previous analyses, the data from Labrie et al. [26] were analyzed according to the invitation-to-screening approach and therefore did not demonstrate a significant reduction in PCa-specific mortality. The CAP trial, meanwhile, reported an incidence rate ratio of 0.96, which was insufficient to support the implementation of PSA screening.

The authors emphasized the substantial heterogeneity among studies, but even though the CAP trial differed markedly from the ERSPC and PLCO trials with a single PSA measurement rather than multiple screening rounds – they concluded that the potential benefits of screening were minimal and should be carefully weighed against short-term harms (biopsies, false positives and negatives) and long-term adverse effects, particularly those related to urinary and sexual function secondary to treatment.

A fourth study, published in 2023, estimated the number of life-years gained through various cancer screening tests, including PSA testing, mammography, fecal occult blood testing, sigmoidoscopy, and colonoscopy [22]. This review included four randomized trials previously analyzed by Ilic et al. [21]. With a median follow-up of 10 years, the estimated gain in life expectancy associated with PSA screening was 37 days (95 % CI: −37 to 73 days), which was not statistically significant.

Unlike the analysis by Ilic et al., the authors of this meta-analysis did not discuss the impact of heterogeneity among the included trials, nor did they update their data to incorporate the 16-year follow-up results published by the ERSPC group in 2019. It is noteworthy that this publication reported a reduction in PCa mortality with a rate ratio of 0.80 (95 % CI: 0.72–0.89; p<0.001), and a decrease in the number needed to invite to prevent one PCa death from 742 at 13 years to 570 at 16 years of follow-up [33].

## To test or not to test? Balancing benefits and harms

The challenges associated with the use of PSA in PCa screening have been repeatedly emphasized. These issues range from its lack of specificity – particularly in older men, in whom benign prostatic hyperplasia often leads to elevated PSA levels – to the risks of overdiagnosis and overtreatment of indolent tumors, as well as the adverse consequences related to diagnostic and therapeutic interventions [34], 35].

All these concerns are highlighted by the authors of the studies analyzed in this review. However, these same studies do not address the potential problem of diagnostic delay that may arise from reducing or eliminating PSA testing in PCa detection. A review that analyzed data from 12,939 patients newly diagnosed with metastatic PCa, obtained from the SEER (Surveillance, Epidemiology, and End Results) database, showed an increase in the number of patients with PCa presenting with metastases between 2004 and 2014, together with a decrease in the age at presentation of disseminated disease [36].

Indeed, the 2018 USPSTF recommendation was less restrictive than that of 2012, indicating that in men aged 55–69 years, screening decisions should be made through shared decision-making between clinician and patient, carefully weighing potential risks and benefits and taking into account individual patient preferences [37].

## Reconsidering the recommendations

Over the past years, several scientific societies and healthcare systems have issued recommendations against the use of PSA measurement for PCa screening. These recommendations have been primarily based on the studies reviewed in this article. The objective of this paper has been, in fact, to highlight the methodological shortcomings of the four main studies evaluated and, consequently, to question the validity of the recommendations opposing PSA-based screening.

The studies included in these reviews show considerable heterogeneity, affecting both the PSA threshold used as an indicator of suspected PCa and the intensity and frequency of screening. Furthermore, these reviews do not adequately account for the lack of equivalence among PSA concentrations measured with different assays, despite recent efforts to achieve greater standardization of results [38], [39], [40].

The positive or negative outcomes of each study may be conditioned by their design and therefore should not be simply aggregated, as done in the aforementioned reviews and meta-analyses. Moreover, the interpretation of the results published by Labrie et al. [26] is debatable due to the high proportion of non-compliant participants. Poor adherence within the group assigned to screening -individuals who were supposed to undergo PSA testing but ultimately did not- dilutes potential benefits of screening in these systematic reviews and meta-analyses. In contrast, Labrie’s study emphasizes the benefit among compliant participants: screening is effective only when it is actually performed.

Additionally, in some cases the trials included in the analyzed systematic reviews and meta-analyses lack sufficient follow-up duration. Indeed, all major recommendations concur that PSA screening should not be offered to men over 70 years of age or, more generally, to those with a life expectancy below 10–15 years [16].

Moreover, despite the importance of follow-up duration, the evaluated systematic reviews and meta-analyses do not always incorporate the most recent updates of previously published trials. For instance, the 2024 study by Bretthauer did not include the 16-year follow-up update of the ERSPC trial, published in 2019. As demonstrated by successive updates of that trial, longer follow-up is crucial to observe a significant mortality benefit from screening, given the slow progression of PCa. A recently published 23-year follow-up update [41] confirms this trend. These results show that the mortality benefit of screening becomes increasingly favorable over time: the absolute risk reduction rises from 0.05 % at 9 years to 0.22 % at 23 years, while the number of subjects needed to invite to screening to prevent one PCa death decreases from 1,919 to 456, and the number needed to diagnose a PCa case falls from 73 to 13.

Similarly, the CAP trial, in its 15-year follow-up update published in 2024 [42], reported a greater reduction in PCa mortality in the screening group than observed in the initial 10-year publication, although the authors still considered this reduction, despite statistical significance, to be modest. In the initial publication with 10 years of follow-up, the relative risk was 0.96 (95 % CI 0.85–1.08, p=0.50), whereas in the 15-year publication it was 0.92 (0.85–0.99, p=0.03).

Furthermore, the authors of the studies reviewed here did not adequately weigh the substantial issue of PSA contamination in the control arm of the PLCO trial, which reached 42–50 % [43], compared to less than 15 % in the ERSPC trial [44]. Indeed, the PLCO investigators themselves acknowledged that their trial should be interpreted as a comparison between an organized screening program and opportunistic screening [45], concluding that their study ultimately does not address the question of PSA screening efficacy for PCa [43].

## The evolving role of PSA in prostate cancer screening

PCa screening using PSA testing has generated extensive debate within the scientific community, with professional societies and health authorities issuing recommendations against its implementation. This article provides a critical evaluation of the main systematic reviews and meta-analyses underpinning these recommendations, identifying key methodological limitations such as heterogeneity among included studies, variability in screening criteria, substantial PSA contamination in the PLCO control arm, and, in some trials, insufficient follow-up duration to adequately assess PCa-specific mortality.

It is important to acknowledge, however, that false positives, as well as overdiagnosis and overtreatment associated with PSA screening, remain significant drawbacks that must be effectively managed. Strategies such as active surveillance – deferring treatment until tumor progression is documented – and the use of novel biomarkers associated with tumor aggressiveness [46] are essential to mitigate these issues.

The recent proposal to use PSA testing more intelligently, as advocated by the European Association of Urology (EAU), involves establishing a baseline PSA measurement and applying risk-stratification strategies -including multiparametric magnetic resonance imaging (MRI) and risk nomograms- to optimize the early detection of aggressive tumors while minimizing overdiagnosis and overtreatment [47], 48]. This personalized approach [49] avoids unnecessary PSA measurements, thereby reducing false positives and related complications, while improving screening quality compared with more indiscriminate protocols.

The adoption of this more rational and individualized screening approach represents an opportunity to enhance the clinical utility of PSA testing, moving beyond the recommendations opposing its use. The European Council’s updated recommendation (December 2022) invited member states to consider organized, population-based PCa screening, specifically proposing PSA testing for men up to 70 years with the use of MRI as a triage test before biopsy [50], 51]. Nevertheless, implementation across member states is heterogeneous and remains largely at the pilot or regional level rather than national scale.

In line with this recommendation, the European Council launched a call to advance PCa screening research, selecting the Prostate cancer Awareness and Initiative for Screening in the European Union (PRAISE-U) project. PRAISE-U, an EU4Health-funded consortium, brings together multidisciplinary teams from multiple countries to evaluate early detection and diagnosis of PCa through harmonized, risk-adapted screening pathways grounded in PSA-based patient stratification within organized programs. This stratification includes three PSA-defined tiers: a low-risk group (PSA <1 µg/L, with long screening intervals), an intermediate-risk group (PSA 1–3 µg/L, prompting closer follow-up), and a high-risk group (PSA >3 µg/L), which triggers first-line risk assessment using validated risk calculators or PSA density before referral for MRI. The pilot centers are in Spain (Galicia and Manresa), Lithuania (Vilnius), Poland (Wroclaw), and Ireland (Dublin). These programs aim to replace unregulated and opportunistic screening by combining PSA-based risk stratification with MRI-guided diagnostic work-up, supported by performance indicators and implementation tools. The overarching goal is to reduce morbidity and mortality while minimizing overdiagnosis through tailored, evidence-driven algorithms and common metrics [52], [53], [54].

Furthermore, several countries and regions have started pilot or regional organized programs that operationalise the Council’s recommendation in real-world settings. Notable examples include Sweden’s organized population-based (OPT) pilots, which invited all 50-year-old men by letter and applied the same PSA-based risk stratification used in PRAISE-U, with a ≥3 µg/L threshold for MRI, followed by PI-RADS and PSA density based biopsy criteria. In the first implementation years, participation reached 35 %, with 2.9 % of tested men showing PSA ≥3 µg/L, 32 % of whom underwent biopsy, yielding 0.39 % clinically significant cancers among all PSA-tested men. These data provide early evidence on feasibility, diagnostic yield, and downstream management patterns [55].

In Germany, the PROBASE randomized trial provides the largest real-world test of a risk-adapted strategy, enrolling 46,642 men aged 45 and assigning them to immediate (PSA value at age 45) vs. delayed (PSA value at age 50) PCa screening. Baseline PSA enabled predefined risk stratification (<1.5, 1.5–2.99, ≥3 µg/L), with multiparametric MRI and selective biopsy recommended only for those with confirmed PSA ≥3 µg/L. In the first screening round, cancer detection was low (48 cases in total, including only 4 ISUP ≥3), and men with PSA <1.5 µg/L (89 % of participants) showed an extremely low 5-year risk of progression triggering further work-up (∼0.06 %), supporting substantially extended screening intervals for this group [56].

Finally, the Lombardy region in Italy launched a digitally enabled, risk-adapted, multilevel pilot activated November 2024 that invites men turning 50 to self-enroll via regional electronic records. The program applies PSA screening followed by MRI and selective biopsy using the same predefined PSA thresholds and stratification tiers as in other European projects [57]. Moreover, other Italian regions -such as Veneto, Emilia-Romagna, and Campania- are already planning to implement comparable organized PCa screening initiatives in order to align with the new European recommendations [58].

These real-world pilots are generating critical operational evidence on participation, MRI demand, biopsy rates, and the positive predictive value for clinically significant cancers. All of these projects consistently implement PSA-based patient stratification, using a PSA cut-off of 3 µg/L to determine eligibility for MRI and, when indicated, biopsy. However, differences in PSA assay methods are not accounted for. For example, Singh et al. [53] and Arsov et al. [56] do not report the methodology used for PSA measurement. In contrast, Bratt et al. [55] note that different reagents are used depending on the region, including Roche Elecsys Total PSA, Siemens Advia Centaur XPT assay, and Siemens Atellica IM PSA assay, but do not mention the potential implications of assay variability on comparability of results.

The Lombardy document is more explicit, providing an appendix with laboratory operational guidance for PSA measurement [57]. It covers pre-analytical variables that may affect PSA levels – ranging from sexual activity and cycling to prostate manipulation – as well as analytical variables that need to be considered, including intra- and inter-assay imprecision, the participation in an external quality control program, and the use of a reference metrological standard. The document also highlights the poor standardization of methodologies and requires participating laboratories to indicate the specific assay method used.

Despite variability and limited standardization in PSA measurement, the Lombardy regional protocol adopts a single cut-off of 3.0 μg/L. This approach acknowledges the potential for a limited number of false positives due to assay differences. The Lombardy document noted that analytical platforms calibrated with Hybritech can produce higher readings compared with WHO-standardized assays and suggested that such differences may affect the choice of cut-offs in diagnostic protocols. It also emphasized the importance of raising physicians’ awareness of these discrepancies, as they could influence clinical decisions.

I agree with the observation regarding potential assay differences in the Lombardy document, but I disagree with the implication that PSA concentrations are systematically lower with WHO-calibrated assays. In the past, some clinical guidelines have suggested this [16]. For example, the Prostate-Specific Antigen Best Practice Statement: 2009 Update by the American Urological Association reported that assays using the 1999 World Health Organization standard yield results 20–25 % lower than those using the Hybritech standard [59]. Similarly, the 2013 European Association of Urology Guidelines on Prostate Cancer stated that a PSA cut-off of 3 or 3.1 µg/L should be considered for World Health Organization–calibrated assays to achieve the same sensitivity and specificity profile found with a cut-off of 4 µg/L [60].

However, published evidence does not consistently support this claim. Stephan et al. [38] reported that PSA serum levels measured with Elecsys (Roche), which uses the WHO standard, were very similar to Hybritech PSA values (Beckman). Similarly, Foj et al. [39] compared several WHO-calibrated assays with Hybritech and found that while Advia Centaur (Siemens) and Architect (Abbott) produced slightly lower PSA levels, Elecsys (Roche), Lumipulse G 1200 (Fujirebio), and Immulite 2000 (Siemens) yielded results closely aligned with Hybritech. Furthermore, Blijenberg et al. [61] reported close agreement between Roche (Cobas), using the WHO calibration, and Beckman assay under Hybritech calibration, with median PSA values of 3.9 and 4.1 µg/L in controls and 3.9 and 4.0 µg/L in PCa patients for Roche and Beckman, respectively.

More recently, Kaufmann et al. [62] assessed multiple WHO-calibrated assays in 76 men and found high correlation of PSA values, but Passing-Bablok analysis revealed substantial differences depending on the assay, with slopes ranging from 0.78 to 1.04. Compared with Cobas 8000 (Roche), PSA values were higher in some assays (e.g., Kryptor, Brahms +9.6 %) and lower in others (e.g., UniCel DxI 800, Beckman Coulter −20.7 %; Architect i1200, Abbott −15.2 %), demonstrating that WHO calibration does not universally result in lower readings.

This variability may impact clinical thresholds and decision-making, highlighting the need for further advances in assay harmonization. PSA assay variability remains an important consideration, because calibration to the WHO standard does not offer equivalent PSA results across different assays. In any case, the recent initiative from the Lombardy regional document for PCa screening, which defines clear requirements for PSA measurement, represents a constructive step toward improving standardization. Clinicians should therefore remain aware of interassay differences, and laboratories should report the specific assay used for PSA measurement.

## Conclusions

In conclusion, the shift towards smarter, risk-based PSA screening with MRI triage–endorsed at EU level is now moving from policy recommendation into practice via consortia-led efforts and regionally implemented pilots. These programs show promise for improving the clinical utility of PSA testing while mitigating harms, but they also emphasize the need for coordinated implementation plans that secure MRI capacity, enforce quality assurance, collect harmonized outcomes, and evaluate equity and cost-effectiveness before widespread national roll-out is undertaken. We must once again stress the need to obtain equivalent PSA results across different platforms. Ensuring analytical comparability is a prerequisite for the safe application of common PSA cut-off values across regions, and therefore for the credibility and scalability of European risk-based screening programs. Only through aligned efforts of health authorities, scientific societies and the *in vitro* diagnostics industry can analytical harmonization be achieved, ensuring that common PSA thresholds adopted in European pilot programs are applied consistently and safely. Building on this alignment, continued collaboration between PRAISE-U, national health authorities and regional pilot sites will be crucial to translate the European recommendation into safe, scalable and evidence-based screening programs.
